# Infants with congenital heart defects have reduced brain volumes

**DOI:** 10.1038/s41598-021-83690-3

**Published:** 2021-02-18

**Authors:** Mikkel B. Skotting, S. F. Eskildsen, A. S. Ovesen, V. S. Fonov, S. Ringgaard, V. E. Hjortdal, M. H. Lauridsen

**Affiliations:** 1 Department of Thoracic and Cardiovascular Surgery, Aarhus University Hospital, Palle Juul-Jensens Boulevard 99, 8200 Aarhus N, Aarhus, Denmark; 2 Center of Functionally Integrative Neuroscience, Department of Clinical Medicine, Aarhus University, Aarhus, Denmark; 3 Montreal Neurological Institute, McGill University, Montreal, QC Canada; 4 MR Research Centre, Aarhus University, Aarhus, Denmark; 5 Institute for Clinical Medicine, Aarhus University, Aarhus, Denmark; 6 Department of Cardiothoracic Surgery, Rigshospitalet, Copenhagen, Denmark; 7 Department of Pediatrics and Adolescent Medicine, Aarhus University Hospital, Aarhus, Denmark

**Keywords:** Neuro-vascular interactions, Heart development, Neurological disorders

## Abstract

Children with congenital heart defects (CHDs) have increased risk of cognitive disabilities for reasons not fully understood. Previous studies have indicated signs of disrupted fetal brain growth from mid-gestation measured with ultrasound and magnetic resonance imaging (MRI) and infants with CHDs have decreased brain volumes at birth. We measured the total and regional brain volumes of infants with and without CHDs using MRI to investigate, if certain areas of the brain are at particular risk of disrupted growth. MRI brain volumetry analyses were performed on 20 infants; 10 with- (postmenstrual age 39–54 weeks, mean 44 weeks + 5 days) and 10 without CHDs (postmenstrual age 39–52 weeks, mean 43 weeks + 5 days). In six infants with- and eight infants without CHDs grey and white matter were also differentiated. Infants with CHDs had smaller brains (48 ml smaller; 95% CI, 6.1–90; *p* = 0.03), cerebrums (37.8 ml smaller; 95% CI, 0.8–74.8; *p* = 0.04), and cerebral grey matter (25.8 ml smaller; 95% CI, 3.5–48; *p* = 0.03) than infants without CHD. Brain volume differences observed within weeks after birth in children with CHDs confirm that the brain impact, which increase the risk of cognitive disabilities, may begin during pregnancy.

## Introduction

Congenital heart defects (CHDs) are among the most common congenital malformations today and affects 0.6–0.8% of infants^1,2^. The reasons for nonsyndromic CHDs remain unclear^3^.

CHDs have been linked to cognitive problems, which are correlated with impairments in several areas, including verbal skill, communication, academic performance, and motor function^4–10^. It has been estimated that between 33 and 43% of children with severe CHDs experience some sort of impaired neurodevelopment^5,11^. These impairments are also present in children with no history of surgical complications or genetic disorders^11,12^.

In children with CHDs, smaller regional brain volumes have been associated with cognitive impairments^10,13^. It has been suggested that the smaller brain volumes could be caused by cerebral circulation disturbances during pregnancy, abnormal cord insertion and other abnormalities of the placenta^9,12,14–22^. Neurodevelopmental disturbances, and thus the susceptibility to neurocognitive impairments, may therefore be present in infants with severe CHDs before any treatment is performed and probably even before birth^6,7,9,23–25^.

CHDs have been associated with hemorrhagic and ischemic brain lesions, predominately in the white matter (WM)^5,6,26–30^ and magnetic resonance imaging (MRI) studies of infants with CHDs have shown both pre- and postoperative brain volume abnormalities^9,12,16,18,25,27,28^. The size of the brain volume reduction varies from 13 to 21%^28,31^.

However, the sample sizes of the cohorts examined with MRI are small, the categories of CHDs are diverse, and the regional distributions of these volume reductions are not consistent from one study to the other^25^. More detailed information on regional brain volume variations may enable the early identification of specific domains of later impairments and possibly allow for earlier intervention.

Therefore, the aim of the current study was to measure regional and total brain volumes in infants with prenatally diagnosed suspected severe CHDs and compare them to age-equivalent infants without CHDs. Furthermore, we screened for abnormalities in the brain as well as hemorrhages and ischemic lesions. The category of severe CHDs used in this article is defined by Hoffman et al.^1^ and includes the majority of the patients who present as severely ill in the newborn period or early infancy.

We hypothesized that the cerebral volumes of the infants with CHDs would be reduced and that we would find regional differences in brain volume, compared to the infants without CHDs. We also hypothesized that infants with CHDs would have more hemorrhages and ischemic lesions.

## Methods

In a prospective cohort study, pregnant women, who were known to each carry a fetus with a CHD that could potentially cause disturbances of the blood flow to the fetal brain, were recruited from Aarhus University Hospital between October 2014 and June 2016. In the same time period, pregnant women who were known to each carry a fetus without a CHD were recruited for comparison. At birth, data regarding the infants’ postmenstrual age, placenta weights, Apgar scores, head circumferences and birth weights are routinely collected, and these data were extracted from the clinical journals. Each infant underwent MRI of the brain in the weeks following birth. Two of the infants with CHDs were scanned prior to surgery. The remaining eight with CHDs was on average scanned 5 weeks (range 6 to 90 days, median 19 days) after the surgical procedure. Additional characteristics are shown in Table 1.

**Table 1:** Characteristics of the infants with CHDs and the infants without CHDs. In the “Males” row, the number of males in each group is shown (percentage). In the other rows, the data are presented as means (standard error). Categorial variables were compared between the infants using the chi-squared test and the Mann–Whitney U test was used for continuous variables. Grams (g), centimeters (cm), magnetic resonance imaging (MRI).

Characteristic	Infants with CHD	Infants without CHD	*P* value
Males	6 (60%)	7 (70%)	0.52
Birth weight (g)	3348 (203.9)	3665 (201.9)	0.18
Birth weight z-scores	− 0.29 (0.31)	0.33(0.25)	0.24
Birth head circumference (cm	34 (0.6)	35 (0.5)	0.08
Birth head circumference z-score	− 0.86 (0.27)	0.13 (0.31)	0.05
Birth weight/head circumference	99 (4.9)	103 (4.5)	0.47
Placenta weight z-scores	− 0.42 (0.3)	0 (0.6)	0.73
Placenta weight (g)	611 (62.8)	710 (46.5)	0.28
Postmenstrual age at birth in weeks + days	39 + 2 (0 + 3)	40 (0 + 4)	0.41
Apgar score at 10 min	9.4 (0.22)	10 (0)	0.06
Postmenstrual age at the MRI scan in weeks + days	44 + 5 (1 + 3)	43 + 5 (1 + 1)	0.62
intracranial hemorrhages and ischemic lesions	6 (60%)	2 (20%)	0.07

### Inclusion and exclusion criteria

The CHDs eligible for recruitment were tetralogy of Fallot, transposition of the great arteries (TGA), coarctation and/or hypoplasia of the aortic arch, hypoplastic left and right heart syndromes, atrioventricular septal defect, and common arterial trunk.

The exclusion criteria were multiple gestation; chromosomal, genetic or multiple abnormalities; the parents spoke a language other than Danish; generally difficult social circumstances, birth prior to postmenstrual week 32, age above 3 months at the time of the postnatal MRI, metal-implants disturbing the MRI, movement artifacts.

### Participants

Informed consent was obtained from parents and/or legal guardians of all subjects. Mothers expecting children with and without CHDs were initially approached at the time of prenatal diagnosis and after obtaining their written and oral consent they were included in a fetal MRI-study^14^. After birth the parents were approached again and if they gave oral and written parental consent, their child was included in the present follow-up study. Sample size estimates were based on results from Ortinau et al.^28^. With a statistical power of 81% and a significance level of 5%, eight participants per group is needed to detect whole brain volumetric differences between CHD and healthy controls.

**Figure 1:**
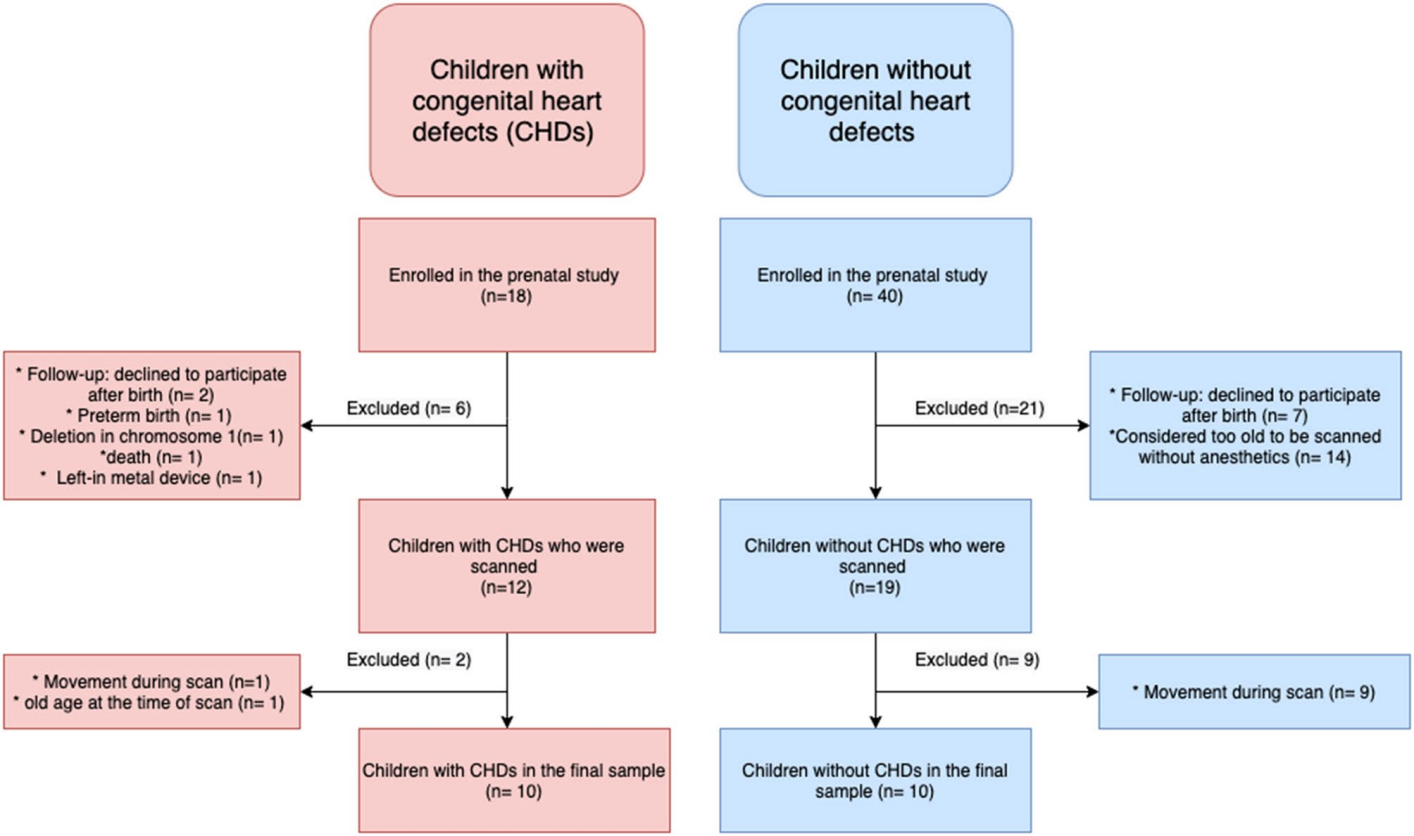
Flow-chart describing the enrollment process.

The final study population consisted of 10 infants (four females) with CHDs and 10 infants (three females) without CHDs. See Fig. 1 for a flow-chart describing the enrollment process.

Five infants had TGA, four had coarctation and/or hypoplasia of the aortic arch, and one had a hypoplastic right heart. A genetic evaluation (CGH array) was performed prenatally with normal results in seven of the included subjects with CHDs and clinical evaluations showed no signs of genetic or syndromic defects in the remaining infants.

### Magnetic resonance imaging (MRI) acquisition

MRI scans were obtained via a Siemens Skyra 3 T (T) system. No anesthetics were used. The infant was fed and wrapped in a MedVac baby Splint®, which is a sleeping bag type of mattress in which the infant was swaddled and held still during the scan^32^. Earplugs and mini-muffs (single-use stick-on earmuffs) were used to moderate the sounds so that the infant could stay asleep during the scan. A pediatric physician (Mette Høj Lauridsen) monitored the infant during the scan and could comfort the baby with a pacifier and 25% glucose. The sleeping baby was placed with the head in a 20-channel transmit/receive head coil.

Initially, a localizer scan was obtained to facilitate the planning. This was followed by an axial 3D MPRAGE T1-weighted (T1w) cerebral scan (acquisition time: 5.5 min, repetition time: 2200 ms, echo time: 2.68 ms, flip angle: 8°, field-of-view: 240 × 240 mm^2^, slice thickness: 1.0 mm, resolution matrix: 256 × 256, reconstruction resolution: 1.0 × 0.94 × 0.94 mm^3^). If the babies were light sleepers, a faster MPRAGE sequence with a 2.5 min acquisition time was used (repetition time: 1900 ms, echo time: 2.36 ms, flip angle 8°, field-of-view: 240 × 240 mm^2^, slice thickness: 1.3 mm, resolution matrix: 224 × 224, reconstruction resolution: 1.3 × 1.07 × 1.07 mm^3^). Four of the 20 infants were scanned using the fast sequence.

Diffusion-weighted, T2-weighted (T2w) FLAIR and T2w susceptibility scans were also performed to detect bleeds, infarcts, and WM lesions as well as structural anomalies. All the scans were described by an experienced MRI physician (Brian Stausbøl-Grøn) blinded to the diagnosis.

### MRI analyses

**Figure 2:**
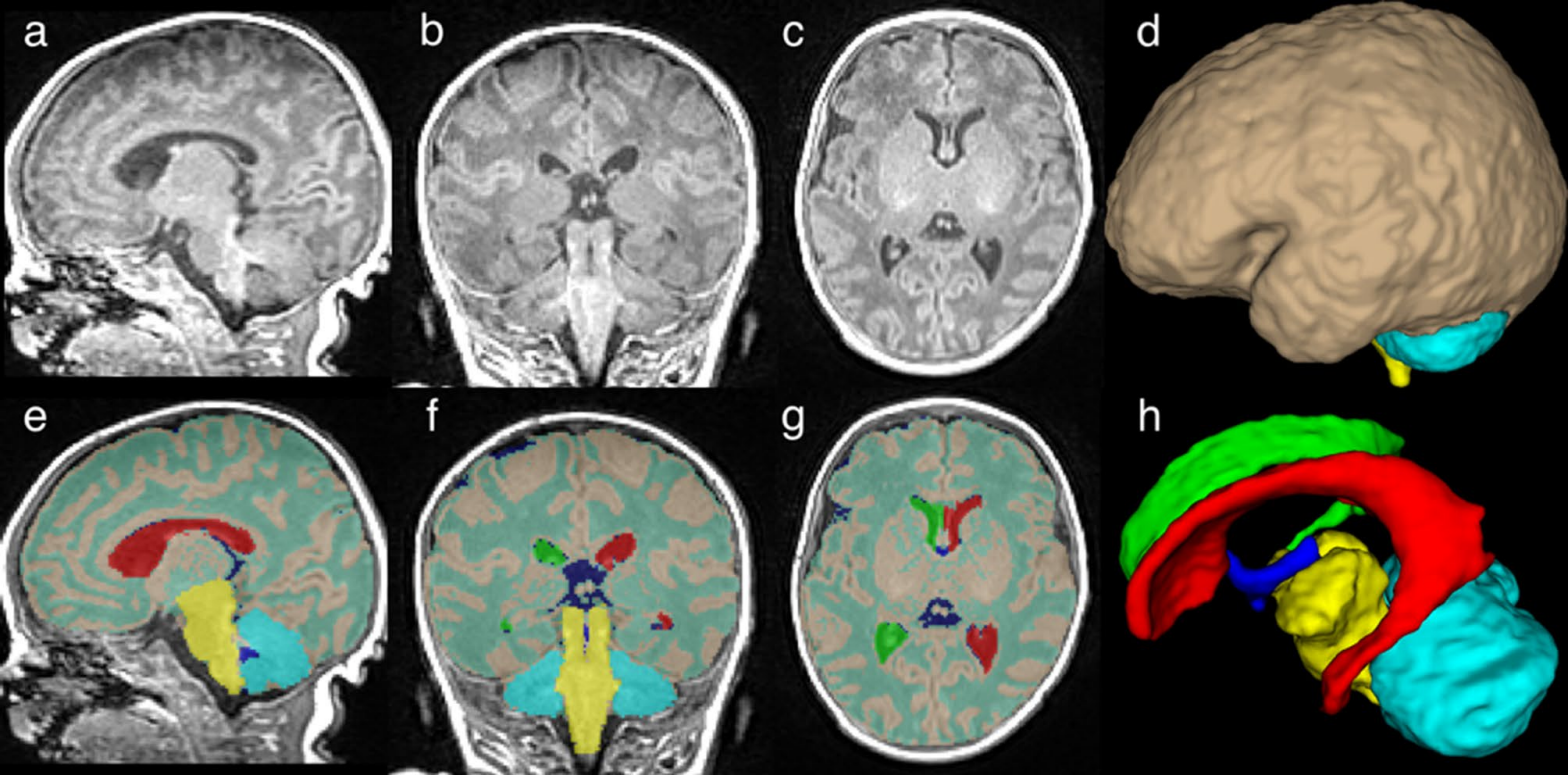
Manually segmented anatomical regions overlaid on a T1-weighted image. T1-weighted image with no overlay in sagittal (**a**), coronal (**b**), and axial (**c**) slices through the volume. Manually segmented anatomical regions overlaid on the same T1-weighted image in sagittal (**e**), coronal (**f**), and axial (**g**) slices through the volume. 3D renderings of the segmentations are shown in (**d**) and (**h**). Red label: left lateral ventricle. Green label: right lateral ventricle. Dark blue: 3rd and 4th ventricles. Yellow: brain stem. Cyan: cerebellum. Turquoise: white matter. Brown: grey matter.

T1w and T2w FLAIR MRI scans were processed using a previously-described framework^33^ and a template in MNI space constructed from 45 three-months-old neonates. The templates were created using the methods described in Fonov et al. 2009^34^ and the images were acquired through the Infant Brain Imaging Study^35^, which can be obtained from The National Institute of Mental Health Data Archive (https://nda.nih.gov/). Ten high-quality T1w images were selected and the following anatomical regions were manually segmented by a trained assistant (Anne Sif Ovesen) using ITK-snap (version 3.6.0-rc1 October 29, 2016)^36^: total brain volume, cerebrum, cerebellum, brainstem, left and right lateral ventricles, and the 3rd and 4th ventricles. The assistant was blinded to the diagnoses. The manually crafted masks were used as training data for automatic segmentations of the entire dataset using multi-resolution patch-based label fusion^37,38^. An example of the segmentations is shown in Fig. 2. All segmentations were visually checked for errors. In addition, the automatic segmentations were cross-validated using the 10 manually labeled images. This was done by automatically segmenting the 10 images leaving out the image under consideration from the training library. The resulting automatic segmentations were compared to the manual segmentations by calculating Dice similarity coefficients (DSC)^39^ and volume-based intraclass correlation coefficients (ICC) (one-way model) ^40^. Cerebral grey matter (GM) and WM were classified using an intensity-based clustering algorithm^41^ on the voxels within the cerebrum mask. Only six of the 10 scans of the infants with CHDs and eight in the infants without CHDs had sufficient quality (visual assessment) for the rater to adequately distinguish between the GM and WM. The remaining scans were therefore not part of the analysis of cerebrum GM and WM. Volumes of the anatomical regions were calculated and values in native scanner space are reported.

### Statistical analysis

Categorial variables were compared between the infants using the chi-squared test and a two-tailed Mann–Whitney U test was used for continuous variables.

A multivariate regression analysis was used to test for differences in total brain and regional brain volumes between the infants with CHDs and the infants without CHDs. The dependent variable y is the volume of the structure and the independent variables × 1 and × 2 are the postmenstrual days and group designation respectively.

Additional multiple regressions were performed in order to ensure that there was no difference between the results of the fast and the slow MRI sequences. The fast and the slow sequences were compared directly and all the regressions concerning brain volumetric was performed with and without the fast sequences. All the regressions were also performed without the newborns that were scanned prior to surgery in order to ensure that this would not affect the results.

We compared our samples to normative data regarding brain volume growth patterns. The normative data of infants was obtained in a Canadian sample through autopsy measurements^42^.

The birth weight z-scores and head circumference z-scores were calculated from a large-population normative dataset^43^. access to reference data granted by the author^43^. The z-scores of the placenta weight were calculated on the basis of normative data from a Norwegian cohort^44^.

All the statistical tests were carried out in Microsoft Excel (Mac version 16.26 2019) with a significance level of 5%. Mean values with 95% confidence intervals (CI) are reported.

### Ethics

The study was approved by the Danish Data Protection Agency (1–16-02–86-14) and by the Central Jutland Regional Committee on Health Research Ethics (journal number 1–10-72–61-14). The protocol of the project conformed to the ethical standards of the Helsinki Declaration of 1975, revised in 2008.

## Results

The gender composition and postmenstrual ages at the time of the MRI scan were similar across infants with and without CHDs (Table 1). The average birth weight/head circumference ratios and placenta weights of both groups were within the normal ranges (≥ 90) ^44,45^. Additional birth characteristics are shown in Table 1.

At birth, the head circumference z-scores of the two groups were different (*p* = 0.05). The head circumference z-score was calculated on the basis of the normative data and adjusted for age and gender^43^. Both groups had z-scores within the normal range^43^ (Table 1).

Cross-validation results for the automatic segmentations are shown in Table 2. Total brain and cerebrum segmentations was in excellent agreement with the manuals segmentations, both in terms of overlap (DSC) and correlation (ICC). The cerebellum and brainstem demonstrated good overlap with the manual segmentations, however, the ICC of these were only moderate. While the DSC of the ventricles were lower than the other structures, these demonstrated ICCs ranging from good to excellent.

**Table 2:** Dice similarity coefficient (DSC) and volume-based intraclass correlation coefficient (ICC) for the automatic segmentations compared to the manual segmentations (n = 10).

Structure	DSC, mean (range)	Intraclass correlation coefficient
Total brain	0.978 (0.969–0.982)	0.962
Cerebrum	0.967 (0.945–0.984)	0.978
Cerebellum	0.897 (0.830–0.933)	0.644
Brainstem	0.911 (0.896–0.926)	0.667
Lateral ventricle, left	0.843 (0.743–0.907)	0.976
Lateral ventricle, right	0.810 (0.649–0.898)	0.937
3rd and 4th ventricles	0.762 (0.683–0.833)	0.773

Results of the regression analysis comparing brain volumes of those with and those without CHDs are shown in Table 3. The total brain volume was, on average, 48 ml smaller (95% CI, 6.1–90, *p* = 0.03) in infants with CHDs compared to infants without CHDs. The cerebrum was, on average, 37.8 ml (95% CI, 0.8–74.8, *p* = 0.04) smaller in the CHD group; when the cerebrum was separated into GM and WM, only the GM was found to be smaller (25.8 ml smaller, 95% CI, 3.5–48, *p* = 0.03). There were no differences between the fast- and the slow MRI sequences and there was no difference when the newborns scanned prior to surgery was not included in the multiple regression.

**Table 3:** Global and regional differences between those with(n = 10) and those without CHDs(n = 10). Cerebral grey matter (GM), cerebral white matter (WM), milliliters (ml).

Region	Volume reduction in the infants with CHDs in ml (standard error)	95% confidence interval in ml	*P* value
Total brain volume	48 (19.9)	6.1 to 90	0.03
GM + WM	37.8 (17.5)	0.8 to 74.8	0.05
Cerebellum	1.6 (1.7)	− 2 to 5.2	0.35
Brainstem	0.8 (0.4)	0 to 1.5	0.05
Left lateral ventricle	0.8 (0.7)	− 0.7 to 2.3	0.30
Right lateral ventricle	0.3 (0.6)	− 0.9 to 1.6	0.59
3rd and 4th ventricles	− 0.1 (0.1)	− 0.4 to 0.2	0.44
GM	25.9 (10)	3.5 to 48	0.03
WM	23.5 (14.1)	− 7.6 to 54.6	0.12

**Figure 3:**
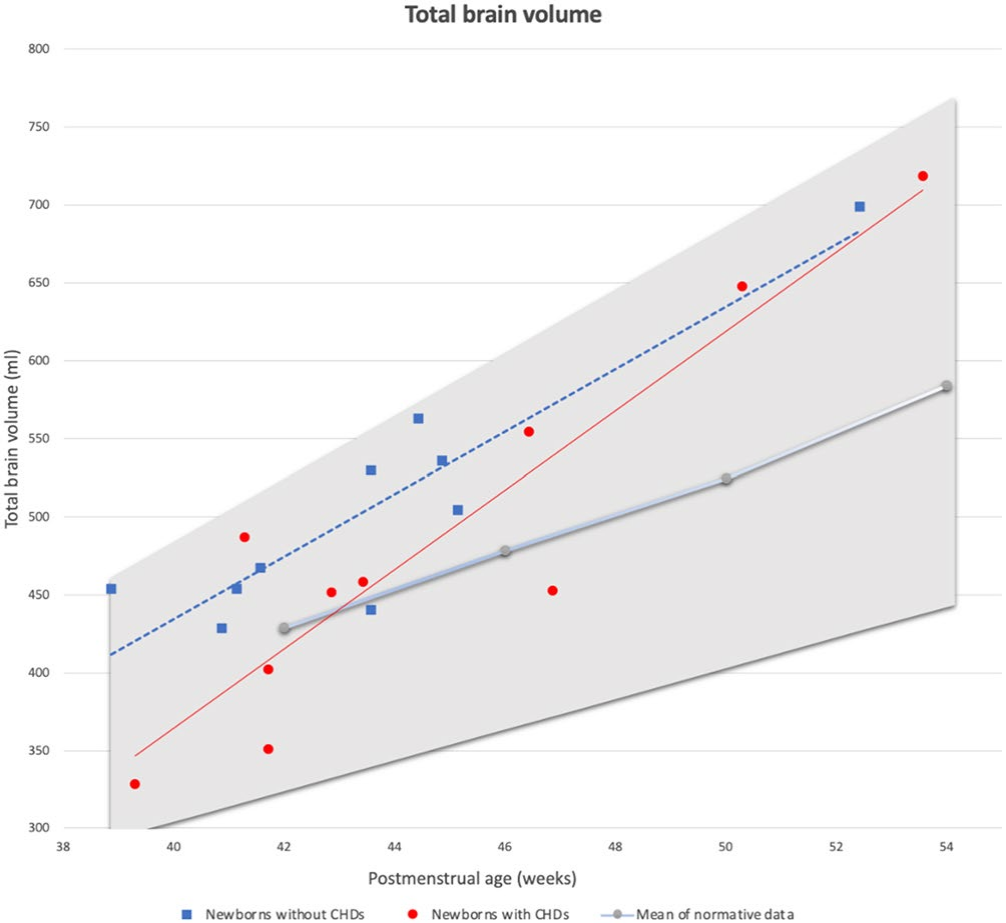
Total brain volume of the infants with congenital heart defects (CHDs) (red dots) and those without CHDs (blue squares). The grey line shows the mean of the reference data and the grey area shows the 95% confidence interval of normative data describing brain volume growth in infants. Milliliters (ml).

The infants with CHDs and the infants without CHDs did not have significantly different brain volumes compared to the normative data. The plotted data of the infants with CHDs, the infants without CHDs, and the mean and 95% confidence interval of the normative data sample are shown in Fig. 3.

When comparing the trendlines of the two groups it appears that the difference in total brain volume was most profound in the youngest group of newborns (gestational age from 39 to 48 weeks). At a postmenstrual age of week 39, the CHD group had an average total brain volume of 339 ml, which is 75 ml (95% CI, 6–90) or 22% smaller than the group with no CHDs, who had an average total brain volume of 414 ml at week 39. At week 52, the regression model predicts a difference in total brain volume of less than one percent. It should be noted, that these observations are based on cross-sectional data in a small sample.

Regarding intracranial hemorrhages and ischemic lesions, we found no differences between the infants with and without CHDs. Small lesions were found in the brains of six infants with and two infants without CHDs. In the infants with CHDs, three infants had small (clinically insignificant) bleeds all grade one. One had a small ischemic lesion in the left thalamus, and one had a punctate WM lesion and a small cerebellar bleed. Finally, multiple small bleeds were found in one infant with a CHD. A follow-up scan two months later disclosed regression of the bleedings; however, multiple cavernous angiomas could not be ruled out. In those without CHDs, one child had small, unspecific parenchymal lesions and one child had a small subdural bleed (with regression of the bleed two months later and no signs of child abuse).

## Discussion

This study revealed smaller brain volumes in infants with CHDs compared to infants without CHDs in our cohort. This is consistent with previous studies^7,9,10,12,15,25,28^.

Our study adds to the existing literature by postnatally characterizing the total, regional, and tissue-specific brain volumes in a single cohort. In a comparable study by Ortinau et al. 15 infants with CHD and 16 healthy controls were scanned at fetal-state and at 3 months of age. This study demonstrated changes in total brain, GM, and cerebellar volumes of infants^28^. We replicated the differences in total brain volume, cerebrum and GM tissue segments, but not in the cerebellum. Ortinau et al. found that infants with CHDs displayed smaller brain volumes over time, indicating slower brain growth in the infants with CHDs. They estimated a total brain volume increase of 11.5 ml per week for infants with CHDs compared to 16.7 ml per week for infants without CHDs from fetal to 3-month MRI^28^. Due to the cross-sectional design, our results cannot reliably estimate brain growth patterns in infants with CHDs and infants without CHDs.

We found no differences regarding intracranial hemorrhages and ischemic lesions between the infants with and without CHDs. Since most of the MRI scans in infants with CHDs were performed after the surgery, it was not possible to determine to what degree these lesions should be attributed to the surgery or if they happened during pregnancy. Since it is not standard procedure to perform an MRI scan on healthy infants, we do not know the prevalence of asymptomatic intracranial hemorrhages. A paper examining this question concluded that mild intracranial hemorrhage is relatively common in late preterm and term infants and mostly represents an incidental finding in clinically asymptomatic babies^46^.

Our study suggests that there is a correlation between CHDs and smaller brain volumes, but the mechanism behind this correlation remains unclear. Therefore, it is difficult to assess to what degree the smaller total brain volume in infants with CHDs should be attributed the fetal development and no other factors, such as effects of surgery. It is however not likely that the brain shrinks secondary to cardiac surgery, but rather that optimizing the hemodynamics will accelerate a compromised brain growth. Nevertheless, the anaesthesia and “cardiac arrest” during surgery could affect the cerebral oxygenation and may negatively affect the brain growth.

It has been suggested that children with CHDs have an increased risk of abnormal cord insertion and other abnormalities of the placenta^20,21^, preterm birth, low birth weight, and fetal growth restriction^47^. This study could not, however, underpin these findings since the infants with CHDs and those without CHDs did not differ in terms of postmenstrual age, placenta weight or birth weight. Another hypothesized contributing factor to the smaller brain volume of infants with CHD is a cerebral oxygenation deficit^9,12,14–19,22^. The hypothesis that tissue hypoxia may be a potential pathogenic factor that possibly affects brain development in fetuses is corroborated in the previous works of Lauridsen et al. Reporting that the fetal cerebral oxygenation of a Danish cohort with- and without CHDs (including the children participating in the present study while they were fetuses) examined using MRI showed significant oxygen deficits in the fetuses with CHDs^14^.

The brain volumes of infants with CHDs were within the 95% confidence levels of the normative data for brain volumes in infants. This could be because the reference data was based on Canadian autopsy data of brain volumes in infants, which could be influenced by both the autopsy technique and underlying causes of death^48^.

### Strengths and limitations

The study consists of infants with and without CHDs who were recruited during the same time period and from the same geographical area. Although the participants in general suffered from less-severe CHDs, compared to most published studies (no participants had hypoplastic left heart syndrome), we still observed smaller brain volumes in those infants with CHDs.

The study is limited by a small and heterogeneous sample of CHDs. Based on results from a previous study, we have sufficient statistical power to detect total brain volume differences between the groups. However, we were unable to estimate the power to detect regional volumetric differences. Therefore, the lack of detected differences at the regional level may be due to type 2 errors, and larger sample sizes may be needed to detect these more subtle differences. Most of the infants with CHD were scanned after cardiac surgery, potentially introducing a number of confounding influences on brain growth.

No Infants expressed phenotypic characteristics that suggested a syndrome and the majority of the infants with CHDs underwent a chromosomal microarray examination. However, an unrecognized genetic predisposition could still influence the results. Since no anesthetics were used, we had to compromise on the MRI scanning time. This meant that some infants were scanned using a faster sequence with lower resolution; however, the regional volumes estimated in the study were relatively large and, thus, had little susceptibility to image resolution issues. Furthermore, the regressions of the volumetric data comparing the fast- and the slow sequence showed no difference. The segmentation accuracy may also affect our results. However, it is reassuring that the cross-validation showed very high Dice overlap and excellent intraclass correlation coefficients between the automatic and the manual segmentations of the structures with significant differences in volume between the two groups (whole brain and cerebrum).

## Conclusion

Our findings indicate that infants with CHDs have smaller brain volumes than infants without CHDs. These changes appear to be region- and tissue-specific. The infants with CHDs had significantly reduced total brain volumes, reduced cerebral volumes, and reduced cerebral GM volumes. These results corroborate previously published data in the field.

## Data Availability

The datasets generated during and/or analysed during the current study are available from the corresponding author on reasonable request.
